# The fusion of two worlds: Non-coding RNAs and extracellular vesicles - diagnostic and therapeutic implications (Review)

**DOI:** 10.3892/ijo.2014.2712

**Published:** 2014-10-17

**Authors:** YUKIE SATO-KUWABARA, SONIA A. MELO, FERNANDO A. SOARES, GEORGE A. CALIN

**Affiliations:** 1 Department of Experimental Therapeutics, The University of Texas M.D. Anderson Cancer Center, Houston, TX, USA; 2 International Research Center, AC Camargo Cancer Center, São Paulo, SP, Brazil; 3 Department of Cancer Biology, The University of Texas MD Anderson Cancer Center, Houston, TX, USA; 4 Institute of Molecular Pathology and Immunology of the University of Porto (IPATIMUP), Porto, Portugal; 5 Department of Stomatology, School of Dentistry, University of São Paulo, São Paulo, SP, Brazil

**Keywords:** microRNAs, extravesicles, exosomes, long non-coding RNA

## Abstract

The role of the extracellular non-coding RNAs, particularly microRNAs present in tumor-derived extravesicles, has been intensively exploited in human cancer as a promising tool for diagnostic and prognostic purposes. Current knowledge on exosomes shows an important role not only as vehicles in the intercellular communication, but the transfer of their content can specifically modulate the surrounding microenvironment, leading to tumor development and progression and affecting therapy response. Based on this, much effort has focused on understanding the mechanisms behind the biology of exosomes and their closely interaction with non-coding RNAs as an efficient tool in tumor diagnostic and therapy. Here we summarize the current knowledge on extracellular and exosomes-enclosed non-coding RNAs, and their importance as potential biomarkers and mediators of intercellular communication in tumor biology.

## 1. Introduction

Data from genome-wide transcriptional analysis in humans have shown that the amount of protein coding transcripts accounts for approximately 2% of the entire genome, while the non-coding RNAs (ncRNAs) correspond to around 98% of all the genomic output (1,2). Interestingly, it has been reported that the proportion of non-coding regions in the genome increases according to the complexity of the organism, suggesting a important role for these sequences in physiology and development of the organisms (3,4). For this reason, much attention has been given to the studies on these non-protein-coding RNAs in many fields, especially in cancer, leading to new hypothesis about cancer biology (5). Additionally, the identification of the circulating microRNAs (miRNAs) in bodily fluids makes them potential non-invasive biomarkers for cancer diagnosis and prognosis.

The comprehension of the mechanisms involved in the interactions between tumor cells and the surrounding environment is relevant for tumor biology elucidation and for the improvement of innovative and more efficient therapy approaches (6). The role of extracellular vesicles in cell-to-cell communication in cancer has been the focus of several studies. MiRNAs are one of the most studied exosomal cargos due to their potential role in tumor diagnosis, prognosis and therapy.

In this review, we summarize the role of ncRNAs in cancer, focusing on miRNAs. Additionally, we focus on the role of exosomes in intercellular communication and their potential use in providing diagnostic opportunities, unraveling new therapeutic targets and predicting therapeutic responses.

## 2. World of non-coding RNAs

The ncRNAs can be divided in two main groups, according to their sizes: long non-coding RNAs (lncRNAs), which are greater than 200 nucleotides and small non-coding RNAs with no more than 200 nucleotides (7,8). These two main categories also show some subgroups, based on the structure and biological function of the transcripts, as long intergenic ncRNAs, pseudogenes, enhancer RNAs, transcribed ultra-conserved region, repeated-associated ncRNAs and antisense RNA in the lncRNAs group. In the small ncRNAs, miRNAs, tiny transcription initiation RNAs, small interfering RNAs, promoter-associated short RNAs, antisense termini associates short RNAs and retrotransposon-derived RNAs have been reported in the literature (5,8).

The miRNAs are the most widely described ncRNA in the literature, since the first small ncRNA lin-4 was described in *C. elegans* more than 20 years ago (9,10). The synthesis of these evolutionarily conserved endogenous short single-stranded RNAs (18–20 nucleotides in length) begins in the nucleus, when the transcription of miRNA-coding genes generate long primary transcript (pri-miRNA) with stem-loop, which will be detached by the RNase III Drosha/Pasha/DGCR8 complex, and then producing a 70-nucleotide precursor (pre-miRNA). After being transported to the cytoplasm by the protein Exportin-5 (XPO5), the pre-miRNA will be converted in mature miRNA by the action of the Dicer and binding to Argonaute 2 (Ago2) to form the RNA-induced silencing complex (RISC) (11,12). Overall, miRNAs regulate gene expression post-transcriptionally, most commonly through the binding to a specific sequence at the 3′-untranslated region (3′-UTR) of a target protein-coding mRNA, causing a translational repression or cleavage of the target transcripts (13). Thus, miRNAs have a relevant role in many pathological and physiological processes, such as cell proliferation, differentiation, development and apoptosis, acting as oncogenes or tumor suppressors, depending on which genes they regulate (14).

The involvement of miRNA genes in cancer was first described in 2002, when the authors reported that two miRNAs (miR-15a and miR-16-1) are mapped at 13q14, a chromosomal region frequently deleted in B-cell chronic lymphocytic leukemia (B-CLL) and that both genes are down-regulated in a high proportion of the cases (15). Since then, the number of studies on miRNAs and cancer has been increasing considerably, adding novel insights into the role of the miRNAs in human tumor such as in hematological malignancies (16–19), colorectal (20–23), breast (24–28), head and neck (29–32) and gastric cancer (33–36).

The lncRNAs are transcripts longer than 200 nucleotides, a cutoff based on RNA purification protocols that exclude small RNAs rather than for its functional role (37). The lncRNAs were first described in a study involving large-scale sequencing and annotation of full-length cDNA libraries in mouse (38), and the number of reports about characterization and functions of the lncRNAs has been constantly increasing in the literature (39). The lncRNAs have many features in common with mRNAs, as transcription by RNA polymerase II, polyadenilation and splicing mechanism. This category of ncRNAs composes a heterogeneous group, which makes the lncRNAs classification difficult (40). Most commonly, the lncRNAs can be classified as sense or antisense, divergent or convergent and intronic or intergenic, depending on their position relative to the neighboring protein-coding genes (7,41). Due to lncRNA structure heterogeneity, it is also difficult to assign a specific function to this group and still requires further studies. Evidences suggest that lncRNAs act mainly in regulation of protein-coding genes transcription, but in more complex ways than the miRNAs (42). lncRNAs can repress the transcription of target genes involving epigenetic modifications like chromatin remodeling, since some lncRNAs have been reported to interact with many chromatin modifiers (43). Additionally, lncRNAs can either play a role as putative gene enhancers or decoy RNAs in transcriptional control (41). Some lncRNAs (antisense ncRNA) also play a role in post-transcriptional regulation by interfering with the RNA splicing mechanism (44).

Due to their roles in the functions mentioned above, the lncRNAs have been related to many human cancers, contributing to tumor development and progress (40). Many lncRNAs have been mapped at cancer risk loci in the human genome, such as PTCSC3 (14q13.3) in thyroid cancer (45,46), PCA3 (9q21–22) in prostate cancer (47,48), ANRIL (9p21) in prostate and breast cancers, leukemia and melanoma (49–52), MALAT1 (11q13) in liver, colorectal, prostate, bladder and lung cancers (53–56).

The role of ncRNAs in many human tumor types has been exhaustingly studied in the past few years and its relevance in mechanisms involved in cancer development and progress is unquestionable. Additionally, the discovery of stable miRNAs in bodily fluids introduced new insights in the ncRNAs comprehension and can represent a new diagnostic approach using less invasive methods (57). The use of circulating miRNAs as tumor biomarkers has many advantages since these transcripts are conserved across species, shows tissue or disease-specific expression and their levels can be quantified by various methods, as microarray profiling, northern blot analysis, *in situ* hybridization, high-throughput sequencing and qRT-PCR, which is the most used method due to its sensitivity, specificity and reliability (58–61). The first evidence of the presence of miRNAs in serum was reported by Lawrie *et al* (2008), who showed the higher serum levels of miR-21 in large B-cell lymphoma (62). Since then, many studies have reported the presence of different circulating miRNAs in various tumor types, such as in colorectum (63,64), esophagus (65,66), breast (67,68), stomach (69,70) and ovary (71,72). Also, the circulating miRNAs have been reported in many other fluids such as plasma, urine, saliva and cerebrospinal fluid (58,73). Considering this discovery, it is possible that other types of ncRNAs similar to lncRNAs can also be identified in bodily fluids (40).

## 3. World of extracellular vesicles

The intercellular communication can occur by a direct cell-to-cell contact including the adhesion junctions, or by the releasing of soluble signaling molecules or by the exchange of cellular fragments as extracellular vesicle (EV) (74,75). The EVs are bilayered membrane vesicles secreted by all cell types, and released in the interstitial space or into circulating bodily fluids, where they can travel long distances until they are up taken by receptor cells (76). Different terminology is used to describe EVs based on their morphology and diameter. Exosomes, microvesicles, ectosomes, microparticles and others, are classified based on their size, shape and membrane surface composition (77). The most accepted classification in the literature shows two major groups of EVs, based on their mechanism of biogenesis and sizes: exosomes and microvesicles (or ectosomes). Additionally, apoptotic bodies have been considered by some as a third category of EVs (78–80). In this review, we will focus on the exosomes and microvesicles.

**Figure 1:**
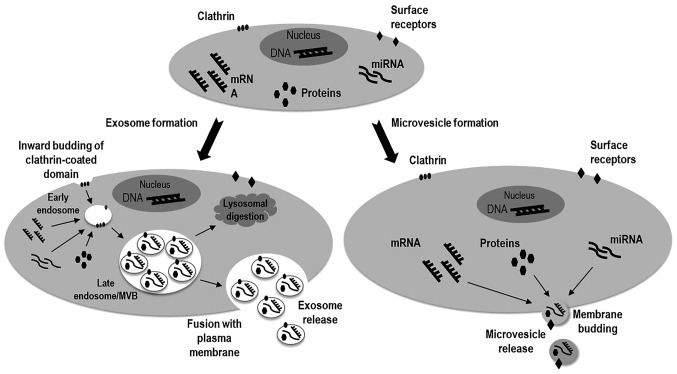
Extravesicles biogenesis by donor cells. Exosomes are originated by the inward budding of clathrin-coated domains in the plasma membrane, generating the multivesicular bodies (MVBs) containing intraluminal vesicles (ILVs) in the late endosome. Later, the MVBs fuse with lysosomes for degradation or with the cell membrane releasing the exosomes to the extracellular space. Microvesicles (MVs) are originated from the plasma membrane through direct outward budding into the extracellular spaces.

Exosomes are 40–140 nm diameter bilayered-membrane vesicles of endocytic origin, with a cup-shaped morphology, showing densities ranging between 1.13–1.19 g/ml (81). The exosomes are originated by the inward budding of clathrin-coated domains in the plasma membrane, generating the multivesicular bodies (MVBs) containing intraluminal vesicles (ILVs) in the late endosome. The formation of ILVs occurs during the endosome maturation, when specific cytosolic proteins are incorporated into these vesicles inside de MVBs. These initial steps occur under control of the ESCRT (endosomal sorting complex required for transport) machinery. Later, the MVBs fuse with lysosomes for degradation or with the cell membrane releasing the exosomes to the extracellular space, process regulated by the RAB family (76,82–84). Microvesicles (or ectosomes) are larger than exosomes, with size ranging between 100 and 1,000 nm in diameter and heterogeneous in morphology. Differently from the exosomes, microvesicles (MVs) are originated from the plasma membrane through direct outward budding into the extracellular space. During this process, the newly originated vesicle captures the donor cellular cytosolic content and the receptors on the plasma membrane (Fig. 1). The regulation of MVs biogenesis is intracellular calcium-dependent and it is the result of the activation of cell surface receptors, phospholipid redistribution and cytoskeletal protein contraction (84,85). The apoptotic bodies (ABs) are membrane vesicles, heterogeneous in shape, showing sizes ranging between 50–500 nm in diameter. The ABs are released from the outward protrusion of the plasma membrane during the late phase of cell death by apoptosis and are featured by the presence of organelles inside the vesicles (85,86).

### The EV cargo specificity

The interaction between the EVs and the target cells can occur by different mechanisms, as direct interaction of the surface proteins of the EVs with the receptors on the target cells, triggering the activation of the intracellular pathways. EVs can also be engulfed by the target cells through membrane fusion or by endocytosis/phagocytosis, with transfer and release of their cargo. Transcripts as mRNAs and miRNAs contained inside de EVs can be transferred to the target cells and be functional (6).

The EVs carry specific contents (cargo) as membrane receptors, ligands, proteins, nucleic acids and infectious agents, depending on the cell of origin and how they were originated from the donor cell (75). There is no consensus regarding the specific content of different EVs, but it seems that MVs are characterized by the presence of cell-surface proteins from the donor cells such as receptors and adhesion proteins. In turn, exosomes have been found to be characterized by proteins associated to their endosomal origin and MVBs formation (84). Some specific markers have been associated to exosomes as tetraspanins (CD9, CD63, CD81 and CD82), major histocompatibility complex class I and II, LAMP1 and LAMP2, flotilins, annexins, TSG101 and heat shock proteins (83,87,88). The protein content of MVs seems to be more heterogeneous, containing cell membrane markers, phosphatidylserine (PS) residues, integrins, selectins and CD40 ligands (84), and high levels of cholesterol and signaling complexes known as lipid rafts (76). Most importantly, it has been shown that the population of exosomes secreted by cancer cells contains a representation of the entire genome of the cell of origin, providing exciting opportunities of using exosomes as a liquid biopsy (89).

### Tumor-derived exosomes

The exosomes are by far the most extensively studied due to their characteristics (as presence in bodily fluids and expression of specific markers) that can contribute not only to intercellular communication but also to their potential role in diagnosis (82). The release of exosomes can be a response to different cellular stress conditions common in cancer, such as hypoxia, acidic pH, heat shock and oxidative stress, resulting in alterations of the tumor microenvironment and distal organs activating angiogenesis and promoting migration and leading to metastasis (90–92).

An important step before considering using the exosomal content for study or diagnosis purposes is a reliable exosome isolation method, to insure the quality of the results. Exosomes can be collected from fluids or cell supernatant by a series of sequential centrifugations to remove larger cellular debris and filtration through 100–220 nm filters to exclude larger EVs, including MVs and apoptotic bodies. Then, the exosomes are pelleted by ultracentrifugation and/or suspension in a sucrose gradient for the completely remove of protein contamination (77,93,94). The exosomes isolation can also be performed using specific filters, immune isolation by magnetic beads or microfluidic separation (95). Recently, some commercial isolation kits are available based on polymer-based precipitation and on the magnetic bead isolation. Then, some additional procedures are necessary to confirm the purity of the isolated exosomes. One of them is to verify the size and shape of the exosomes by electron microscopy analysis. Vesicles diameter and morphology can also be assessed by specific instruments that can visualize, characterize and measure small vesicles. Another important factor that must be evaluated is the protein content, that can be assessed by flow cytometry and western blot analysis for markers as CD63, CD81, Tsg101 and flotilin (77,96).

## 4. The fusion of the two worlds

### Exosomal miRNAs

In the bodily fluids, the miRNAs have been reported to play a role at intercellular communication, and can act at short and long distant sites in a hormone-like behavior (14,87). The transport of circulating miRNAs can be carried by protein transporters or by exosomes. It is known that serum contains ribonucleases, suggesting that the circulating miRNAs are protected from the RNase action within extravesicles. The miRNA recruitment to the exosomes depends on the attachment of RNA-induced silencing complexes (RISCs) to the ESCRT components. However, the release of exosomal miRNAs is under control of a ceramide-dependent machinery, as reported by Kosaka *et al* (73). These authors showed that the inhibition of neutral sphingomyelinase 2 (a regulator of the ceramide biosynthesis) resulted in lower levels of miRNA secretion (73).

The first evidence of the existence of miRNAs in exosomes was reported by Valadi *et al*, showing that these vesicles contain both mRNA and miRNAs, which can be transferable to another cell, where the transcripts can be functional (97). Since then, the number of studies regarding the identification of exosomal miRNAs in cancer has been increasing in the literature. A summary of some studies in the literature in this field is in Table I. Most of these reports are based on *in vitro* studies involving a variety of cancer cell lines, identifying the exosomal miRNA content as in breast cancer (98,99), leukemia (100), melanoma (101,102), prostate cancer (103), ovarian (104) and gastric cancer (105). The transcripts content of the exosomes usually can differ from that in the donor cells, and the exosomal miRNA profile of tumor cells can also differ from that released by normal controls (106).

Studies have reported evidence of the intercellular transfer of the exosomal content between different cells. Chiba *et al* showed that exosomes derived from colorectal cancer cells can be transferred to hepatoma and lung cancer cells (107). In addition, some of these reports have demonstrated that the transferred exosomal content can be functional in the receptor cells. Yang *et al* reported the presence of a specific miRNA for IL-4-activated macrophage - miR-233, in the co-cultured breast cancer cells and it can enhance the invasiveness potential of the receptor cells (108). The transfer of the leukemia cell line-derived exosomal miR-92a to the endothelial cells affected the endothelial cell migration and tube formation in the receptor cells (109). Kosaka *et al* showed that exosomal miR-143 derived from a normal prostate cell line act as a tumor suppressor by inhibiting the growth in the target prostate cancer cells (110). Similar results were reported by Roccaro *et al* in a study demonstrating that the exosomal miR-15 from the normal bone marrow mesenchymal stromal cells causes a tumor suppressor effect when transferred to tumor cells, where this miRNA is downregulated (111). In a recent study, Valencia *et al* demonstrated that the exosomal miR-192 derived from lung adenocarcinoma cell lines repressed the angiogenic activity in the co-cultured endothelial cells by the inhibition of the proangiogenic factors (112). More recently, Zhou *et al* showed that the transfer of exosomal miR-105 to non-metastatic breast cancer cells induces metastasis and vascular permeability by targeting the cellular tight junction protein ZO-1 (113).

The intercellular transfer of the exosome cargo can also affect the resistance or sensitivity of cancer cells to therapy. The transfer of the exosomes derived from chemoresistant breast cancer cell lines can also spread the resistance potential to receptor chemosensitive cell lines, possibly due to the action of the exosomal content as miR-100, miR-222 and miR-30a (114). Similarly, in lung cancer, Xiao *et al* reported that after miR-21- and miR-133-enriched exosome transfer from the chemoresistant tumor cells, the chemosensitive target cells acquire resistance to the drug exposure (115).

It is well known that hypoxia is an important factor that triggers angiogenesis and metastasis formation and evidence has been presented for the involvement of the exosome in this mechanism. King *et al* found an increased concentration of released exosomes and higher expression of exosomal miR-210 secreted by breast cancer cells under hypoxic conditions when compared to normoxic cells (116). In leukemia, the miR-210 can also be found in a subset of miRNAs upregulated in exosomes released by tumor cells under hypoxic conditions (117).

The exosomal miRNA expression profiling from serum and plasma samples has also been assessed in glioblastoma, where the expression of 11 miRNAs known to be upregulated was slightly lower in exosomes than in the donor cells but still reflecting the tumor profile (118). When the serum samples from ovarian cancer patients were evaluated, a distinct exosomal miRNA profile was identified from that of benign disease (119). Another study reporting a potential use of exosomal miRNAs as diagnostic markers showed a higher expression of miR-21 released from esophageal cancer serum samples when compared to non-tumoral samples, and it correlated with advanced tumor stages, lymph node involvement and metastasis (120). Exosomal let-7a, miR-1229, miR-1246, miR-150, miR-21, miR-223 and miR-23a from colorectal tumor samples and cancer cell lines are more highly expressed than those from healthy controls samples and normal colon cell lines, and the expression levels of these miRNAs are significantly decreased in exosomes samples collected after tumor resection, indicating the cancer status (121).

The exosomal miRNA profiling from plasma samples was assessed to develop a diagnostic screening method for lung adenocarcinoma. The expression pattern of 12 specific upregulated miRNAs (miR-17-3p, miR-21, miR-106a, miR-146, miR-155, miR-191, miR-192, miR-203, miR-205, miR-210, miR-212, miR-214) in tumor samples was similar in the tumor plasma-derived exosomes and distinct from the control samples, indicating exosomal miRNAs could be relevant as a screening method for this tumor (122). In another report, the exosomal miRNAs miR-378a, miR-379, miR-139-5p and miR-200-5p were identified as possible markers to distinguish tumor from normal samples in lung adenocarcinoma (123). In addition, the miRNAs miR-151-5p, miR-30a-3p, miR-200b-5p, miR-629, miR-100 and miR-154-3p are possible markers to discriminate lung adenocarcinoma from granulomas (123). More recently, Rodríguez *et al* evaluated the exosomes derived from bronchoalveolar lavage (BAL) and plasma samples from lung cancer, in which the exosomal miRNA content derived from tumor plasma samples is more elevated than in the BAL, suggesting that the a higher concentration of exosomal miRNAs are released in the plasma than in the bronchoalveolar fluid (124).

The use of exosome as a diagnostic tool has also been evaluated in other fluids than plasma and serum samples. Recently, a report identified the exosomal miRNAs in bile from cholangiocarcinoma patients and a potential diagnostic panel that includes miR-486-3p, miR-16, miR-1274b, miR-484 and miR-191 as predictive markers (125). In a study involving cervicovaginal lavage fluids, the miR-21 and miR-146a were highly expressed in fluids from cervical cancer samples when compared to those from HPV(+) and HPV(−) normal samples (126).

### Exosomal lncRNAs

The study of lncRNAs is a relatively new field on cancer research, and many questions about their expression and functions remain unclear, like the presence of these ncRNAs in the bodily fluids. Since the use of circulating miRNAs in diagnostic screening methods and therapeutics have been intensively evaluated in many tumor types, the presence of released lncRNAs in bodily fluids, specially within extravesicles as exosomes, could be a source of novel potential biomarkers for diagnosis, prognosis and therapeutics purposes. Of our knowledge, there are only few data about this particular aspect of the lncRNAs (Table II). The ucRNA (ultranconserved lncRNA) TUC399 was identified in exosomes derived from hepatocellular cancer cell lines, and the intercellular transfer of exosomal TUC399 can contribute to tumor growth and progression (127). More recently, the same group demonstrated that the expression of lncRNAs linc-RoR (long intergenic non-coding RNA, regulator of reprogramming) in hepatocellular cancer is responsive to hypoxic conditions and the transfer of exosomal linc-RoR can modulate the intercellular response to hypoxia (128).

At present, reports regarding the circulating lncRNAs in bodily fluids are also scant in the literature. In a study evaluating the expression of the lncRNAs H19, HOTAIR and MALAT1 in gastric cancer plasma samples, Arita *et al* (2013) showed that only H19 is higher expressed in tumor samples than in the controls, and reported significanly decreased expression levels in post-operative tumor samples, indicating that the release of lncRNAs into the plasma can reflect the disease status (129). In another report, Ren *et al* identified the MALAT1-derived mini-RNA (MD-miniRNA) as potential novel plasma biomarker in prostate cancer (130). Some reports have demonstrated the use of lncRNA PCA3 as a specific and reliable marker detectable in urine samples from patients of prostate cancer, instead of the standardized use of the prostate-specific antigen (PSA). The evidence that highly upregulated in liver cancer (HULC) lncRNA expression is significantly higher in plasma tumor samples than in the healthy controls indicates the use of this lncRNA as potential circulating biomarker for diagnosis in hepatocellular cancer (131,132). The evaluation of lncRNAs expression in plasma samples from leukemia and multiple myeloma showed that TUG1, MALAT1, HOTAIR and GAS5 are more highly expressed in leukemia than in the control samples, and only lincRNA-p21 is upregulated in multiple myeloma (133).

However, some limitations in using exosomal ncRNAs in diagnostics have been pointed out by many authors. The specificity and sensitivity of exosomal tumor marker detection in bodily fluids is still challenging. For example, serum and plasma-derived extravesicles as exosomes can be released by other than tumor cells, such as different blood cell types, affecting the purity of the tumor-derived exosome samples. In addition, the release of these exosomes depends on the age of the patient, infection or inflammation status of the disease, possibly introducing a bias in comparison analysis if not appropriately normalized to these conditions. Another issue that must be considered is the need of a standardized protocol for collecting and handling of the samples, as well as the exosomes isolation method (95,106).

### Relevance in therapy

Since the miRNA is able to target multiple genes and signaling networks simultaneously, acting like oncogenes or tumor suppressor factors, it makes them a suitable tool for therapeutics interventions. A well and highly specific design is necessary for a successful result and to prevent undesirable targets. However, one of the principal limitations of this approach is the nuclease activity, causing the degradation before the miRNAs can achieve the targets. The use of vesicles for the delivery of exogenous therapeutic molecules to the targets has been intensively considered as a new promising therapeutic intervention. As mentioned before, the exosomes have the ability to transfer functional proteins and transcripts as perfect non-immunogenic carriers of therapeutic agents to target cells, making them suitable as therapeutic tool (134).

Considering that the exosome content can act as modulator of the microenvironment, facilitating tumor growth and metastasis, the blockade of the production, release and uptake by receptor cells could reverse the influence of the increased levels of exosomes in tumor progression (95). Based on this, focusing on the inhibition of key components of the extravesicle production and release, such as the members of the ESCRT machinery, could be a useful strategy for therapy (6).

A third possible direction is represented by the drug or gene delivery by extravesicles to the target sites. Considering the elucidation of the intercellular transfer by exosomes, many reports have demonstrated the use of the extravesicles as small RNA carriers (135,136). Intercellular transfer by exosomes can be used as miRNA carriers to restore miRNA expression in the target cells, where they play a therapeutic role as tumor suppressor factors. The targeted delivery of miRNAs by exosomes was demonstrated in a study in breast cancer cells expressing high levels of EGFR. This was achieved by the engineering of the donor cells to modify the surface of the exosomes to express the transmembrane domain of the PDGFR fused to the GE11 peptide. Then, the modified exosomes can deliver the let-7a miRNA after intravenous injection to EGFR-expressing xenograft breast cancer tissue in immunodeficient mice (137). The ability of the miRNAs to target multiple genes can be a limitation for the specificity of this method as a selective approach for targeted therapy. The use of synthetic siRNA has been exploited as a more selective therapeutic tool. In an interesting study reported by Alvarez-Erviti *et al*, dendritic cells expressing a specific protein of the exosomal membrane Lamp2b fused to a neuron-penetrating RVG peptide were isolated from mice, and the exosomes derived from these cells were loaded with exogenous siRNA to GAPDH by electroporation. Subsequently, these RVG-targeted exosomes were intravenously injected, which delivered GAPDH siRNA to specific cells in the brain, leading to a selective gene knockdown (138). Similarly, another report showed the delivery of siRNA into monocytes and lymphocytes by exosomes as gene delivery vector, reflecting a selective gene silencing of MAPK1 (139).

## 5. Concluding remarks

The discovery of the intercellular communication by the extravesicles has opened a new field for tumor biology. Exosomes can be found in the bodily fluids in a variety of tumor types and many reports have proved that the exosomal content as proteins, mRNA, miRNA and DNA can reflect the disease status, making them suitable for biomarkers for non-invasive diagnostic and prognosis purposes. With the advance of the engineering that permits the manipulation of the exosomal content and surface markers, many studies have been focusing on the development of therapeutic approaches in various tumor types, involving more specific delivery to the target tumor cells with more selective and efficient results. However, despite the efforts focusing on the study of the extracellular vesicles, specially exosomes, there are many aspects of the their biology that still need to be elucidated so that it would improve the advantages of the use of this promising approach in tumors.

## Figures and Tables

**Table I:** Summary of the studies reporting the identification of exosomal miRNAs in cancer.

Tumor	Sample	Exosome extraction	miRNA	Refs.
Breast cancer	Cell line	SC, 0.22 μm filtering and UC	miR-233	(108)
Breast cancer	Cell line	SC and UC or ExoQuick (System Biosciences)	miR-210	(116)
Breast cancer	Cell line	SC, 0.22 μm filtering and UC	miR-100, miR-17, miR-222, miR-342-3p, miR-451, miR-30a	(114)
Breast cancer	Cell line	SC, 0.22 μm filtering, UC and sucrose gradient	miR-198, miR-26a, miR-34a, miR-49a, let-7a, miR-328, miR-130a, miR-149, miR-602 and miR-92b	(99)
Breast cancer	Serum samples, tumor samples, cell line, animal models	SC, UC	miR-105	(113)
Cervical cancer	Cervicovaginal lavage fluid	SC and UC	miR-21 and miR-146a	(126)
Cholangiocarcinoma (biliary tree)	Bile sample	SC and 0.22 μm filtering	miR-222, miR-126, miR-486-3p, miR-484, miR-19a, miR-19b, miR-16, miR-191, miR-31, miR-1274b, miR-618, miR-486-3p, miR-16, miR-1274b, miR-484, miR-191	(125)
Colorectal cancer	Cell line	SC and 0.22 μm filtering	miR-21, miR-192, miR-221	(107)
Colorectal cancer	Serum samples	SC, 0.22 μm filtering and UC	let-7a, miR-1229, miR-1246, miR-150, miR-21, miR-223 and miR-23	(121)
Esophageal cancer	Serum samples	SC, 0.45 μm filtering and ExoQuick (System Biosciences)	miR-21	(120)
Gastric cancer	Cell line	SC, 0.1 μm filtering and UC	let-7 family (a, b, c, d, e, f, g, i)	(105)
Glioblastoma	Tumor samples, serum samples	SC, 0.22 μm filtering and UC	let-7a, miR-15b, mR-16, miR-19b, miR-21, miR-26a, miR-27a, miR-92, miR-93, miR-320, miR-20	(118)
Glioblastoma	Tumor samples, cell lines, animal models	SC, 0.22 μm filtering, and UC	miR-1	(140)
Leukemia	Cell line	ExoQuick (System Biosciences)	miR-19a, miR-146-5p, miR-454, miR-18b, miR-574-3p, miR-21, miR-431, miR-345, miR-210, miR-197, miR-20a, miR-24, miR-19b, miR-130b, miR-106b, miR-224, miR-210, miR-652, miR-379, miR-185	(117)
Leukemia	Cell line	SC, 0.22 μm filtering and ExoQuick (System Biosciences)	miR-17–92 cluster, miR-24, miR-222	(109)
Leukemia	Cell line	SC, 0.22 μm filtering and UC	miR-1908 and miR-298	(100)
Lung adenocarcinoma	Plasma samples	Size exclusion by chromatography, magnetic beads (EpCAM)	miR-17-3p, miR-21, miR-106a, miR-146, miR-155, miR-191, miR-192, miR-203, miR-205, miR-210, miR-212, miR-214	(122)
Lung adenocarcinoma	Plasma samples	ExoQuick (System Biosciences)	miR-378a, miR-379, miR-139-5p, miR-200-5p, miR-151-5p, miR-30a-3p, miR-200b-5p, miR-629, miR-100 and miR-154-3p	(123)
Lung adenocarcinoma	Cell line	SC, 0.22 μm filtering, and UC	miR-192	(112)
Lung cancer	Cell line	UC and ExoQuick (System Biosciences)	miR-21, miR-98, miR-133b, miR-138, miR-181a, miR-200c	(115)
Lung cancer	Plasma sample, bronchoalveolar lavage fluid	SC, 0.22 μm filtering, and UC	miR-222, miR-126, miR-144, miR-302a, miR-302c	(124)
Melanoma	Cell line	SC, 0.22 μm filtering and UC; SC and ExoQuick (System Biosciences)	miR-181b, miR-181a, miR-4802-3p, miR-23b, miR22, miR-107, miR-103a, miR-9, miR-338-3p	(101)
Melanona and colon carcinoma	Cell line	SC, 0.22 μm filtering, prominin-1 based immuno-magnetic selection	miR-216b, miR-889, miR-4307, miR-4272, miR-203, miR-4289, miR-3149, miR-203, miR-3145, miR-1911, miR-513a-3p, miR-3916, miR-886-3p, miR-1182, miR-3613-5p, let-7i, miR-3132, miR-3914, miR-3618, miR-1307, miR-3614-3p, miR-519c-3p, miR-3160, miR-3153, miR-4278, miR-3646, miR-3926, miR-515-5p, miR-3169, miR-590-3p, miR-525-5p, miR-548g, miR-365, miR-525-3p, miR-320d	(102)
Multiple myeloma	Cell line	0.22 μm filtering, UC and ExoQuick (System Biosciences)	miR-125q-3p, miR-128, miR-15a, miR-185, miR-192, miR-212, miR-324-3p, miR-331-5p, miR-345, miR-422a, miR-429, miR-511, miR-576-3p, miR-618, miR-9, miR-1271, miR-139, miR-148, miR-151-3p, miR-15b, miR-19b-1, miR-21, miR-34b, miR-378, miR-589, miR-592, miR-625, miR-93	(111)
Ovarian cancer	Serum samples, cell line	Magnetic activated cell sorting (MACS) - EpCAM	miR-21, miR-141, miR-200a, miR-200c, miR-200b, miR-203, miR-205, miR-214	(119)
Ovarian cancer	Cell line	SC, UC and sucrose gradient	let-7	(104)
Prostate cancer	Cell line	SC, 0.22 μm filtering, and UC	miR-143	(110)
Protaste cancer	Cell line	SC and UC	miR-1280, miR-720 and miR-1260b	(103)

SC, sequential centrifugation; UC, ultracentrifugation; MV, microvesicle.

**Table II:** Summary of the reports of the circulating lncRNAs in cancer.

Tumor	Sample	Extravesicles isolation	Long ncRNA	Refs.
Gastric cancer	Plasma samples, cell lines	NA	H19, HOTAIR, MALAT1	(129)
Hepatocellular cancer	Cell line	UC and density gradient separation	TUC399	(127)
Hepatocellular cancer	Tissue samples, plasma samples	NA	HULC	(132)
Hepatocellular cancer	Cell line, animal model	SC and UC	linc-RoR	(128)
Leukemia and multiple myeloma	Plasma samples	NA	TUG1, MALAT1, HOTAIR, lincRNA-p21, GAS5	(133)
Prostate cancer	Tissue samples, plasma samples	NA	MALAT-1 and PCA3	(130)

SC, sequential centrifugation; UC, ultracentrifugation; NA, not applied.
